# High-Dimensional Immune Profiling of Human Retinal Detachment Samples Using Spectral Flow Cytometry: A Protocol for Intraocular Immunotyping

**DOI:** 10.3390/mps8060141

**Published:** 2025-11-20

**Authors:** Laura Molinero-Sicilia, Alejandro G. del Hierro, Nadia Galindo-Cabello, Pablo Redruello-Guerrero, Salvador Pastor-Idoate, Ricardo Usategui-Martín, David Bernardo

**Affiliations:** 1 Unit of Excellence Institute of Applied Ophthalmobiology (IOBA), University of Valladolid, 47011 Valladolid, Spain; laura.molinero24@uva.es (L.M.-S.); nadiaregina.galindo@uva.es (N.G.-C.); predruello@saludcastillayleon.es (P.R.-G.); 2 Department of Cell Biology, Genetics, Histology, and Pharmacology, Faculty of Medicine, University of Valladolid, 47003 Valladolid, Spain; 3 Networks of Cooperative Research Oriented to Health Results (RICORS), National Institute of Health Carlos III (ISCIII), 28040 Madrid, Spain; 4 Mucosal Immunology Lab, Institute of Biomedicine and Molecular Genetics from Valladolid (IBGM, University of Valladolid-CSIC), 47003 Valladolid, Spain; alejandro.hierro.gonzalez@uva.es (A.G.d.H.); david.bernardo@uva.es (D.B.); 5 Department of Ophthalmology, Hospital Clínico Universitario de Valladolid, 47003 Valladolid, Spain; 6 Centro de Investigaciones Biomédicas en Red de Enfermedades Infecciosas (CIBERINFEC), 28029 Madrid, Spain

**Keywords:** spectral flow cytometry, retinal detachment, intraocular immunophenotyping, leukocyte profiling, vitreous, retina, immune markers, high-dimensional cytometry, gating strategy, ocular inflammation

## Abstract

Retinal detachment (RD) disrupts the eye’s immune-privileged status, causing a local inflammatory response that contributes to adverse clinical outcomes, including proliferative vitreoretinopathy and suboptimal visual recovery. Comprehensive profiling of intraocular immune cells will offer mechanistic insights and support the development of personalized immunomodulatory strategies. Here, we describe a robust and standardized protocol for the collection and high-dimensional analysis of the intraocular immune infiltrate from patients undergoing RD surgery, using state-of-the-art spectral cytometry. Vitreous and retinal tissue samples were obtained during standard surgical procedures, without the need for additional invasive interventions. Our approach integrates two complementary protocols: one that enables selective isolation of immune cells by sorting for CD45^+^ populations, and a second one that applies a 39-color spectral cytometry panel to profile the general landscape of immune subpopulations. The panel can identify up to 62 distinct viable immune subsets per sample, along with their functional status, as it includes expression of 13 functional markers. Hence, we hereby detail sample preparation, staining, and acquisition workflow, as well as the gating strategy and essential steps necessary for reproducible immunophenotyping. Our protocol, which enables high-dimensional immune profiling from minimal biological material, provides a valuable platform for studying ocular inflammation in RD and other retinal diseases.

## 1. Introduction

The retina is a highly specialized neural tissue of the eye and a functional extension of the central nervous system. Thus, it is located within the immune-privileged environment of the eye, which actively limits inflammation to preserve visual function. This privilege is maintained by both structural barriers, mainly the blood–retinal barrier, and a series of local immunoregulatory mechanisms [1]. However, retinal detachment (RD), a condition characterized by the separation of the neuroretina from the retinal pigment epithelium, breaches both the anatomical and immunological integrity of the retina. While the reduction in visual acuity in patients with RD is primarily due to photoreceptor (PR) apoptosis, this cell death represents only one consequence of a complex degenerative process. 

The detachment triggers a neuroinflammatory cascade that promotes neurodegeneration, leading to poor visual outcomes and multiple clinical complications. Several molecular pathways are rapidly activated, including upregulation of inflammatory cytokines such as IL-6, IL-8, IL-1B, and TNF-α, along with activation of resident microglia and Müller glial cells. Oxidative stress and mitochondrial dysfunction further contribute to PR apoptosis. Additionally, the detachment impairs metabolic exchange between the retina and choroid, causing hypoxia and nutrient deprivation that sustain and amplify the degenerative environment [2]. 

Although surgical techniques have significantly improved over the years and anatomical reattachment is achieved in more than 90% of cases, functional visual recovery often remains incomplete [3,4,5]. Preclinical research aimed at understanding the mechanisms underlying RD has primarily focused on PR apoptosis and retinal neurodegeneration. Nevertheless, despite the central role played by the ocular immune system following RD, less attention has been given to the immune mechanisms that may influence these degenerative processes and post-surgical recovery, hence limiting our understanding of the disease and the development of targeted immunomodulatory therapies that could improve clinical outcomes. This can be explained because the retina is a delicate tissue, and in the context of RD, the number of recoverable cells can be extremely limited since access to human retinal tissue is minimal. Such low cellularity, combined with the fragility of intraocular samples, makes detailed cellular characterization of human immune response challenging [6].

Spectral flow cytometry is an advanced form of conventional flow cytometry that records the entire emission spectrum of each fluorochrome rather than detecting light only at fixed wavelengths. This improves signal resolution, allows better separation of overlapping fluorochromes, and enables the simultaneous analysis of many more markers [7]. These advantages make it especially useful for studies with very limited sample availability, since it maximizes the information obtained from minimal cell numbers. Indeed, this methodology is already being successfully implemented in animal models, especially in mice, and has helped reveal important aspects of the immune response in RD [8,9,10,11]. However, there is still no standardized protocol for processing and analyzing human intraocular samples, making it difficult to apply these findings to clinical practice. Moreover, most studies focus solely on the retina, often overlooking that the immune response in the eye involves more than one component [9,12]. This is particularly relevant in conditions such as RD, which often result from vitreous traction. In such cases, many immune cells that infiltrate the site of injury can reside in the vitreous and would be missed if only the retinal tissue were analyzed. Moreover, while the retina often contains resident immune and neural cells that contribute to local inflammation, the vitreous acts as a reservoir for soluble molecules and infiltrating immune cells [12]. Thus, analyzing both tissues provides a more comprehensive and accurate representation of the ocular immune environment.

We provide an optimized and reproducible protocol for the simultaneous collection and high-dimensional analysis of immune cells from both retinal tissue and vitreous fluid. Importantly, both samples can be collected during RD surgery, without the need for additional invasive procedures. This makes the method highly practical and ethically appropriate in clinical research. Two different but complementary protocols have been optimized. The first one allows the enrichment of viable singlet leukocytes infiltrating both compartments, allowing for targeted downstream analyses. The second one, built up from the Optimized Multicolor Immunofluorescence Panel [13], introduces a modified 39-marker spectral cytometry panel capable of identifying up to 62 immune cell subsets, together with the expression of 13 functional markers to address the phenotype and function of each subset. Our approach provides a powerful tool for characterizing immune mechanisms during neurodegeneration after RD. By enabling comprehensive and reproducible characterization of the intraocular immune landscape in RD, this protocol may help identify patient-specific inflammatory signatures that could guide the development of tailored immunomodulatory therapies aimed at improving visual outcomes.

## 2. Experimental Design

**Figure 1:**
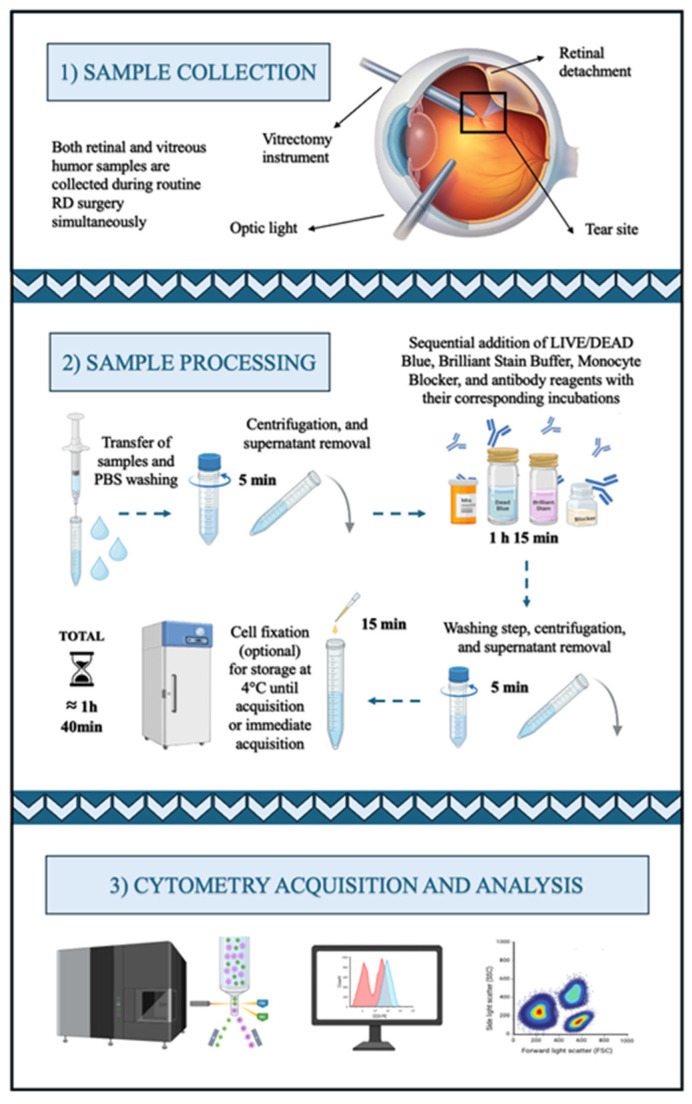
Graphical summary of the general workflow. The estimated total time shown for the sample processing corresponds to the second proposed protocol, designed for spectral flow cytometry acquisition. The first protocol for spectral cell sorting is shorter, with an approximate total processing time of 1 h.

Figure 1 illustrates the overall experimental workflow, detailing each step and the estimated time required to complete it. Retinal and vitreous humor samples were collected simultaneously during routine RD surgery. Retinal and vitreous humor samples were collected simultaneously during routine RD surgery. All surgeries were conducted under sterile conditions in the operating room by the same experienced vitreoretinal surgeon to ensure consistency across procedures. Retrobulbar anesthesia was administered using diluted 5% lidocaine, and a 25-gauge vitrectomy was performed with the Constellation Vision System^®^ (Alcon^®^, Geneva, Switzerland).

After placing three trocars in the pars plana, a central core vitrectomy was performed before opening the infusion to collect undiluted vitreous. The sample was obtained using very high cut rates (20,000 cuts/min) and low vacuum (150–200 mmHg) to ensure a gentle, continuous aspiration stream that minimizes traction on the detached retina and iatrogenic tears risk. Approximately 0.5–1 mL of vitreous was carefully aspirated directly into a sterile syringe connected to the vitreous cutter, stopping aspiration as soon as the vitreous cavity began to collapse, to prevent exerting traction or accidentally engaging the detached retina. The sample was immediately transferred to sterile tubes and kept on ice. After this, a complete central and peripheral vitrectomy was performed to release any residual vitreoretinal traction. 

Retinal tissue samples were then obtained by gently lifting and excising the free retinal flap located at the edge of the retinal tear using the vitrectomy cutter set to 800 cuts/min, taking care to minimize mechanical trauma. The free retinal flap at the edge of the tear was collected by aspirating it directly through the vitrectomy cutter (vitreotome) port, after removing all vitreous traction around the tear. The vitreous cutter itself was then used to gently lift and excise the flap. An illustrative demonstration of this procedure is available in Supplementary Video 1 of the study by Galindo-Cabello et al. (2025) [14]. All samples were promptly placed in sterile tubes and transported on ice from the operating room to the laboratory to preserve tissue integrity until processing. Then, washing the sample with phosphate-buffered saline (PBS) is essential to remove residual debris and unbound reagents, ensuring a clean cell suspension for accurate staining and analysis. Additionally, for viscous samples, especially vitreous humor, washing decreases viscosity, making centrifugation easier in the following steps. 

Once the samples are washed and the cells are in suspension, they are first stained with the LIVE/DEAD Blue viability stain. This stain specifically identifies viable cells within the sample, which is crucial for analysis, especially given the high incidence of cell death in retina and vitreous samples. Subsequently, Brilliant Stain Buffer Plus is added to minimize nonspecific interactions and enhance staining quality. True-Stain Monocyte Blocker is further applied to reduce Fc receptor-mediated nonspecific binding, particularly on monocytes and macrophages. Each reagent addition is followed by vortexing and incubation steps to ensure adequate mixing and optimal binding.

For the first protocol, a brief incubation with a CD45-specific antibody is sufficient to isolate and gate immune cells within the sample. Thus, the total estimated time for sample processing is reduced to approximately 1 h. In contrast, the second, longer protocol uses a cocktail of 37 additional antibodies (38 in total; see Table 1), enabling comprehensive immunophenotyping of the samples.

### 2.1. Materials

1. Phosphate-Buffered Saline (PBS) (Cytiva, Marlborough, MA, USA, Cat. no: SH30256.01); stored at room temperature (RT).
2. Bovine Serum Albumin (BSA) (Gibco, Waltham, MA, USA, Cat. no: 30063-572); stored at 4 ºC.
3. NaN3 (Sigma-Aldrich, St. Louis, MO, USA, Cat. no: S2002-25G); stored in a dry place at RT.
4. Wash Buffer (manually prepared; see reagent setup for details).
5. LIVE/DEAD Fixable Dead Cell Stain (Invitrogen, Waltham, MA, USA, Cat. No: L34962); stored at 4 °C in the dark.
6. Brilliant Stain Buffer Plus (BD Biosciences, Franklin Lakes, NJ, USA, Cat. no: 566385); stored at 4 °C protected from light.
7. True-Stain Monocyte Blocker (BioLegend, San Diego, CA, USA; Cat. no: 426103); stored at 4 °C in the dark.
8. 10% Neutral Buffered Formalin (Fisher Scientific, Waltham, MA, USA, Cat. no: 316-155); stored at RT in a tightly closed container.
9. Paraformaldehyde (PFA) 1% (manually prepared; see reagent setup for details)
10. Fluorescently labeled antibodies (see Table 1)

### 2.2. Equipment

- Cytek Aurora™ cell sorter (CS System) 3-laser (Cytek, Fremont, CA, USA)
- 5-laser Cytek^®^ Aurora (Cytek, Fremont, CA, USA)

### 2.3. Software

- SpectroFlo^®^ acquisition software v.3.2.1 (Cytek, Fremont, CA, USA)

OMIQ Data Science Platform for high-dimensional cytometry analysis (© Omiq, Inc. 2022, Boston, MA, USA) or any similar software analysis

## 3. Procedure


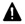
 All centrifugation steps should be performed at 4 °C to minimize cell loss, which is especially critical in this protocol, as the number of cells recovered from intraocular samples is often low.

All centrifugations are performed at 500× *g* for 5 min at 4 °C.

### 3.1. Retina and Vitreous Humor Samples Processing for Spectral Cell Sorting

1. Transfer each sample individually into separate cytometry tubes.
2. Add 1 mL PBS and, if necessary, perform mechanical dissociation using a vortex or pipetting up and down.
3. Centrifuge and carefully discard the supernatant, leaving approximately 100 μL of residual volume.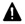 **CRITICAL STEP:** The vitreous sample is viscous; therefore, the first washing step should be performed carefully to avoid cell loss.
4. Add 5 µL LIVE/DEAD™ Blue (1:40 dilution) and vortex. Incubate for 15 min at room temperature (RT) in the dark.
5. Wash with 1 mL of Wash Buffer and centrifuge.
6. Discard the supernatant as step 3.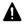 **CRITICAL STEP:** This washing step should remove most of the vitreous viscosity. Additional washes before the antibody incubation steps are generally not recommended to avoid cell loss.
7. Add 10 µL Brilliant Stain Buffer Plus and vortex.
8. Add 5 µL True-Stain Monocyte Blocker and vortex.
9. Add 2.5 µL anti-CD45 PerCP antibody and vortex. Incubate for 20 min at RT in the dark.
10. Add 1 mL of Wash Buffer and centrifuge.
11. Discard the supernatant, leaving approximately 300 µL of cell suspension.
  **OPTIONAL** STEP: If cell functionality is not necessary for subsequent experiments, samples can either be acquired fresh or fixed for later acquisition. However, fixation is recommended to ensure consistency across all samples. To fix, add 300 µL 1% PFA and vortex. Incubate for 10 min RT in the dark. Add 1 mL Wash Buffer, centrifuge, and discard the supernatant, leaving approximately 300 µL of cell suspension.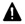 **CRITICAL STEP:** Use a 100 µm filter if the samples cannot be totally dissociated to prevent clogs in the flow cytometer and ensure accurate acquisition.
12. Store at 4 °C until acquisition, preferably within 48 h.

### 3.2. Retina and Vitreous Humor Samples Processing for Spectral Cytometry Acquisition

1. Transfer each sample individually into separate cytometry tubes.
2. Add 1 mL PBS and, if necessary, perform mechanical dissociation using a vortex or by pipetting up and down.
3. Centrifuge and carefully discard the supernatant, leaving approximately 100 μL of residual volume.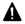 **CRITICAL STEP:** The vitreous sample is viscous; therefore, the first washing step should be performed carefully to avoid cell loss.
4. Add 5 µL LIVE/DEAD™ Blue (1:40 dilution) and vortex. Incubate for 15 min at RT in the dark.
5. Wash with 1 mL of Wash Buffer and centrifuge at 500× *g* for 5 min at 4 °C.
6. Discard the supernatant as step 3.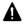 **CRITICAL STEP:** This washing step should remove most of the vitreous viscosity. Additional washes before the antibody incubation steps are generally not recommended to avoid cell loss.
7. Add 10 µL Brilliant Stain Buffer Plus and vortex.
8. Add 5 µL True-Stain Monocyte Blocker and vortex.
9. Add 2.5 µL of anti-IgG BV605 antibody and vortex. Incubate for 10 min at RT in the dark.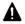 **CRITICAL STEP:** Do not wash after this step.
10. Add 1.25 µL anti-TCRγδ PerCP/eFluor 710 antibody and vortex. Incubate for 10 min further at RT in the dark.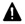 **CRITICAL STEP:** Do not wash after this step.
11. Add 1.25 µL anti-CXCR5 BV750 and 2.5 µL anti-CCR5 BV563 antibodies and vortex. Incubate for 10 min further at RT in the dark.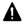 **CRITICAL STEP:** Do not wash after this step.
12. Add 100 µL of the modified OMIP-069 antibody mix and vortex. Incubate for 30 min further at RT in the dark.
13. Add 1 mL of Wash Buffer and centrifuge.
14. Discard the supernatant, leaving approximately 300 µL of cell suspension.
  **OPTIONAL STEP**: If cell functionality is not necessary for subsequent experiments, samples can either be acquired fresh or fixed for later acquisition. However, fixation is recommended to ensure consistency across all samples. To fix, add 300 µL 1% PFA and vortex. Incubate for 10 min RT in the dark. Add 1 mL Wash Buffer, centrifuge, and discard the supernatant, leaving approximately 300 µL of cell suspension.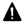 **CRITICAL STEP:** Use a 100 µm filter if the samples cannot be totally dissociated to prevent clogs in the flow cytometer and ensure accurate acquisition.
15. Store at 4 °C until acquisition, preferably within 48 h.

## 4. Expected Results

**Figure 2:**
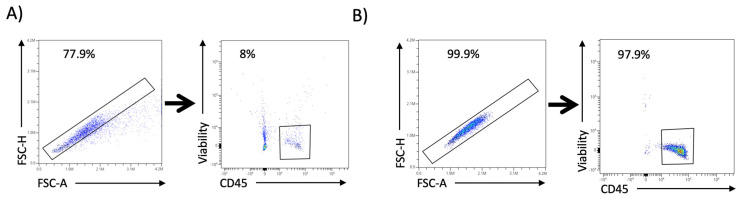
A gating strategy was followed for the identification of viable leukocytes in retina and vitreous humor samples in spectral sorting. (**A**) The first plot shows singlet gating. The second plot displays the viable leukocytes gate obtained from the Singlets gate (indicated by the arrow). (**B**) The same plots from the same sample were derived from the re-acquisition of the viable leukocyte fraction of Figure 2A.

**Figure 3:**
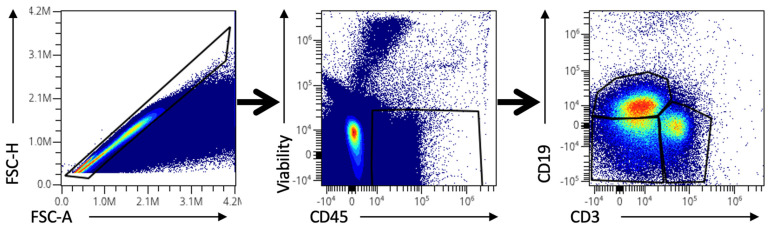
A gating strategy was followed for the identification of cell populations within viable leukocytes in retina and vitreous humor samples in spectral flow cytometry. A total of 54 samples (27 retinas and 27 vitreous humor) are overlaid in each plot.

**Figure 4:**
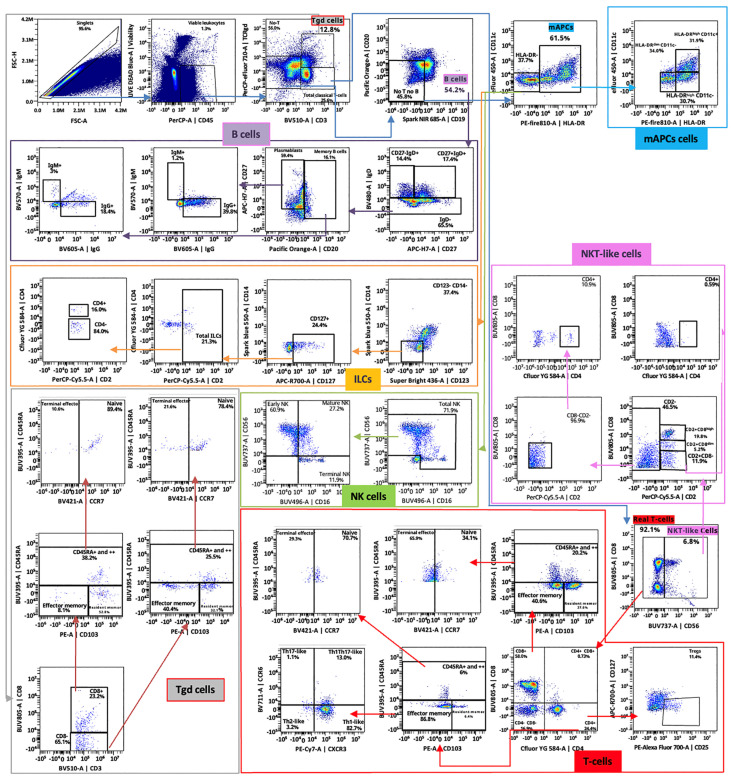
Identification of different immune cell subsets by spectral cytometry. The figure shows the hierarchical gating strategy, the main populations identified, and their subsets.

The protocols described herein constitute robust, reproducible tools for detecting immune cells in retinal and vitreous humor samples. To the best of our knowledge, it represents the first one that allows the processing of these human samples for high-dimensional cytometry analysis. The cytometry plots presented below (Figure 2, Figure 3 and Figure 4) serve as representative examples of the gating strategies and immune cell population distributions achievable using this protocol. Notably, these plots are illustrative only and do not reflect aggregated or statistically analyzed data. Single-cell isolation was ensured by gating singlets using forward and side scatter parameters, excluding debris and doublets, and selecting only viable cells.

### 4.1. Spectral Cell-Sorter Enrichment

Viable CD45^+^ leukocytes were isolated and efficiently enriched following the protocol described in Section 3.1. Figure 2 shows two pair cytometry plots obtained from the spectral sorter acquisition of one retina sample. In Figure 2A, the first plot shows the Singlets gate, using forward scatter height (FSC-H) and forward scatter area (FSC-A), which account for 77.9% of the total events. Viable leukocytes are extracted from this gate, accounting for 8% of the Singlet events, based on viability and CD45^+^ (Figure 2A). Thus, viable leukocytes are 6.23% of the total events in this sample. This viable leukocyte gate can be sorted into a cytometry tube to be acquired again, a process known as post-sort enrichment. This enrichment was successful, as the gates above accounted for more than 99% of events, as shown in Figure 2B.

### 4.2. Spectral Cytometry Analysis

With the application of the 38-antibody panel described in Section 3.2, high-dimensional characterization of immune population distributions within retinal and vitreous humor samples was successfully achieved. The number of cell subsets that can be confidently identified depends critically on the total cell count in each sample, as successive gating steps may reduce accuracy when the total cell count is low. A minimum of 50 cells in each parental population is recommended to reliably identify the corresponding subpopulations through successive gating steps. Thus, special care in washing and the overall experimental processing procedure is essential for an optimal efficiency of the method. Despite this limitation, our methodology allows for a general overview of the main immune populations in any properly processed sample. Markers that identify the major immune populations are particularly robust and useful, even in samples with low total cell counts. These include CD3, CD4, CD8, and TCRγδ for T cells; CD56, HLA-DR, CD14, and CD16 for NKT-like cells, myeloid antigen-presenting cells, and NK cells; and CD20, CD19, and IgD for B cells. Since these populations are often abundant and well-defined, these markers are less sensitive to individual variability or limitations in cell number that may affect successive gating.

Figure 3 shows three cytometry plots with an overlay of several retina and vitreous humor samples. Singlets and viable leukocyte gates are like the ones displayed in Figure 2. The third plot reveals the three main populations found within viable leukocytes in these samples: CD3^+^ (lymphocytes and NKT-like cells), CD19^+^ (B cells), and CD3-CD19^−^ (NK cells, myeloid antigen-presenting cells, etc.).

To explore the full potential of this approach, Figure 4 shows the gating strategy designed to identify up to 62 different immune cell subsets per sample. Importantly, this level of resolution can only be achieved when the total cell count of the sample is sufficiently high. In addition, for each cell subset, our implementation of the OMIP069 multi-color panel (10.1002/cyto.a.24213) enables the identification of 13 different functional markers (CCR5, CCR6, NKG2A, NKG2D, CXCR5, PD1, CD57, CD103, CTLA4, CD95, CXCR3, HLA-DR, and VISTA), allowing functional profiling.

This approach generates data that supports both the quantification of immune population frequencies and in-depth functional profiling, making it a valuable tool for both basic and translational research. Our methodology addresses two significant technical limitations regarding RD research: the need to avoid additional invasive procedures (since both retina and vitreous samples are collected during routine surgery) and the limited volume of viable intraocular sample obtained per patient (as our high-dimensional panel is optimized for small samples). Importantly, interindividual variability in cell yield and subset distribution may arise from factors such as the extent and chronicity of RD, patient age, or surgical manipulation. Standardizing surgical sampling procedures, processing times, and staining conditions is therefore essential to minimize this variability and ensure reproducibility and comparability across samples. Importantly, this protocol is primarily intended for research purposes rather than routine clinical application due to its technical requirements, specialized equipment, and associated costs. Nevertheless, it provides a solid foundation for future adaptations using smaller, targeted panels that could make intraocular immunotyping more feasible in clinical settings.

In the context of RD, where inflammation plays a central role in retinal neurodegeneration and poor visual recovery, this methodology offers unprecedented resolution for simultaneously analyzing the immune landscapes of both retinal and vitreous compartments. As such, it constitutes a powerful tool for advancing our understanding of neuroinflammatory processes in human RD. Moreover, this workflow is adaptable for characterizing other retinal and vitreous diseases and supports the study of more targeted and personalized immunotherapeutic strategies in the eye.

## 5. Reagents Setup

1. Wash Buffer: prepared fresh every week with the following composition: 500 mL PBS, 1 g BSA, and 0.445 g NaN_3_. Stored at 4 °C and used within one week.
2. PFA 1%: prepared by mixing 90% PBS with 10% neutral buffered formalin; stored at 4 °C and used within one month of preparation.
3. Modified OMIP-069 antibody mix: prepared based on the panel described by Park LM et al. [13], with slight modifications. Our version includes 38 antibodies instead of the original 39. CD337, CD24, CD39, and CD159c-specific antibodies are omitted, and VISTA, CD103, and CTLA4 ones are added. For the spectral cytometry protocol (see Section 3.2), the antibody mix used in step 15 contains all antibodies that were not pre-incubated individually (anti-IgG BV605, anti-TCRγδ PerCP/eFluor 710, anti-CXCR5 BV750, and anti-CCR5 BV563). Thus, a total of 34 antibodies are combined as indicated in Table 1.

## Figures and Tables

**Table 1:** List of the 38 anti-human monoclonal antibodies used for high-dimensional immunophenotyping, including instructions for mix preparation (dilutions and respective volumes referred to a 100 µL mix).

Specificity	Clone	Fluorochrome	Supplier (Cat. No)	Purpose	Dilution	Volume (mL)/Test
CD1c	L161	Alexa Fluor 647	BioLegend (331510)	Dendritic cells, NKT-Like cells	1/20	5
NKG2A (CD159a)	REA110	APC	Miltenyi (Bergisch Gladbach, Germany) (130-113-563)	NK, NKT-Like, and γδ T cell activation/differentiation	1/50	2
CD38	HIT2	APC/Fire810	BioLegend (356644)	Monocyte, dendritic cell, T cell, and B cell activation/differentiation	1/40	2.5
CD27	M-T271	APC/H7	BD Biosciences (560222)	T and B cell differentiation	1/20	5
CD127	HIL-7R-M21	APC/R700	BD Biosciences (565185)	Cytokine receptor; T cell differentiation	1/20	5
CD141	1A4	BB 515	BD Biosciences (565084)	Dendritic cell differentiation	1/80	1.25
CD45RA	5H9	BUV 395	BD Biosciences (569489)	T cell differentiation	1/80	1.25
CD16	3G8	BUV 496	BD Biosciences (612944)	Monocyte, NK cell, and dendritic cell differentiation	1/160	0.625
CCR5 (CD195)	2D7/CCR5	BUV 563	BD Biosciences (741401)	Chemokine receptor; Monocyte, dendritic cell, T cell, and B cell differentiation	1/20	5
NKG2D (CD314)	1D11	BUV 615	BD Biosciences (751232)	NK cell differentiation	1/20	5
VISTA	TU66	BUV 661	BD Biosciences (750502)	Immune checkpoint	1/50	2
CD56	NCAM16.2	BUV 737	BD Biosciences (612766)	Pan NK cell, γδ T cell activation	1/80	1.25
CD8	SK1	BUV 805	BD Biosciences (612889)	T cell, NK, and NKT-Like cell lineage	1/160	0.625
CCR7 (CD197)	G043H7	BV 421	BioLegend (353208)	Chemokine receptor; T cell differentiation	1/20	5
IgD	IA6-2	BV 480	BD Biosciences (566138)	B cell differentiation	1/160	0.625
IgM	MHM-88	BV 570	BioLegend (314518)	B cell differentiation	1/32	3.125
IgG	IA6-2	BV 605	BD Biosciences (566138)	B cell differentiation	1/40	2.5
CD3	SK7	BV510	BioLegend (44828)	Pan T cell, NKT-Like cells	1/20	5
CD28	CD28.2	BV 650	Bio Legend (302946)	T cell and NK cell differentiation	1/40	2.5
CCR6 (CD196)	G034E3	BV 711	BioLegend (353436)	Chemokine receptor; T cell and B cell differentiation	1/80	1.25
CXCR5	RF8B2	BV 750	BD Biosciences (747111)	Chemokine receptor; T cell differentiation	1/80	1.25
CD279 (PD-1)	EH12.2H7	BV 785	BioLegend (329930)	T cell inhibitory receptor	1/20	5
CD4	SK3	cFluor YG584	Cytek (R7-20041-100T)	T and NKT-Like cell lineages	1/200	0.5
CD11c	3.9	eFluor 450	eBioscience (Santa Clara, CA, USA) (48-0116-42)	Pan myeloid lineage	1/20	5
CD57	HNK-1	FITC	BioLegend (359604)	NK and CD8^+^ T cell immune senescence	1/80	1.25
CD20	HI47	Pacific Orange	Invitrogen (MHCD2030)	Pan-B cells	1/20	5
CD103	REA205	PE	BioLegend (350206)	Integrin for tissue-resident cells	1/100	1
CD25	CD25-3G10	PE/Alexa 700	Life Technologies (Carlsbad, CA, USA) (MHCD2524)	IL-2 receptor	1/40	2.5
CD95 (Fas)	DX2	PE/Cy5	BioLegend (305610)	Cytotoxicity marker	1/160	0.625
CXCR3 (CD183)	G025H7	PE/Cy7	BioLegend (353720)	Chemokine receptor; Dendritic cell, T cell, and B cell differentiation	1/20	5
CTLA4	P30-15	PE/Dazzle 594	BioLegend (369616)	Immune checkpoint	1/50	2
HLA-DR	L243	PE/Fire 810	BioLegend (307683)	T cell and monocyte activation, NK cell lineage discrimination, and dendritic cell marker	1/20	5
CD45	HI30	PerCP	Invitrogen (MHCD4531)	Pan leukocytes	1/40	2.5
CD2	TS1/8	PerCP/Cy5.5	BioLegend (309226)	NK cell differentiation	1/20	5
TCRyg	B1.1	PerCP/eFluor710	Thermofisher (Waltham, MA, USA) (46-9959-42)	Pan γδ T cell	1/80	1.25
CD14	63D3	Spark Blue 550	BioLegend (367148)	Monocyte differentiation	1/40	2.5
CD19	HIB19	Spark NIR 685	BioLegend (302270)	Pan B cells	1/80	1.25
CD123	6H6	Super Bright 436	eBioscience (62-1239-42)	Il-3 receptor	1/40	2.5

## Data Availability

The original contributions presented in this study are included in the article. Further inquiries can be directed to the corresponding authors.

## Abbreviations

The following abbreviations are used in this manuscript:

BSA	Bovine Serum Albumin
OMIP	Optimized Multicolor Immunofluorescence Panel
PBS	Phosphate-Buffered Saline
PFA	Paraformaldehyde
PR	Photoreceptor
RD	Retinal Detachment
RT	Room Temperature

## References

[1] Katamay R., Nussenblatt R.B., Ryan S.J., Sadda S.R., Hinton D.R., Schachat A.P., Wilkinson C.P., Wiedemann P. (2013). Chapter 27-Blood–Retinal Barrier, Immune Privilege, and Autoimmunity. Retina.

[2] Redruello-Guerrero P., Gómez-Tomás M., Rechi-Sierra T., Molinero-Sicilia L., Galindo-Cabello N., Usategui-Martín R., Pastor-Idoate S. (2025). Inflammatory Mechanisms in the Management and Treatment of Retinal Detachment. Metabolites.

[3] Park D.H., Choi K.S., Sun H.J., Lee S.J. (2018). Factors associated with visual outcome after macula-off rhegmatogenous retinal detachment surgery. Retina.

[4] Di Lauro S., Castrejón M., Fernández I., Rojas J., Coco R.M., Sanabria M.R., Rodríguez de la Rua E., Pastor J.C. (2015). Loss of Visual Acuity after Successful Surgery for Macula-On Rhegmatogenous Retinal Detachment in a Prospective Multicentre Study. J. Ophthalmol..

[5] Lai M.M., Khan N., Weichel E.D., Berinstein D.M. (2011). Anatomic and Visual Outcomes in Early versus Late Macula-On Primary Retinal Detachment Repair. Retina.

[6] Basu S., Hassman L., Kodati S., Chu C.J. (2024). Intraocular Immune Response in Human Uveitis: Time to Look Beyond Animal Models. Am. J. Ophthalmol..

[7] Tabor S.J., Yuda K., Deck J., Gnanaguru G., Connor K.M. (2023). Retinal Injury Activates Complement Expression in Müller Cells Leading to Neuroinflammation and Photoreceptor Cell Death. Cells.

[8] Xiao W., Shahror R.A., Morris C.A., Caldwell R.B., Fouda A.Y. (2023). Multi-Color Flow Cytometry Protocol to Characterize Myeloid Cells in Mouse Retina Research. Bio. Protoc..

[9] Maidana D.E., Gonzalez-Buendia L., Pastor-Puente S., Naqvi A., Paschalis E., Kazlauskas A., Miller J.W., Vavvas D.G. (2023). Peripheral Monocytes and Neutrophils Promote Photoreceptor Cell Death in an Experimental Retinal Detachment Model. Cell Death Dis..

[10] Tang Y., Xiao Z., Pan L., Zhuang D., Cho K.-S., Robert K., Chen X., Shu L., Tang G., Wu J. (2020). Therapeutic Targeting of Retinal Immune Microenvironment with CSF-1 Receptor Antibody Promotes Visual Function Recovery After Ischemic Optic Neuropathy. Front. Immunol..

[11] Lin W., Wang P., Qi Y., Zhao Y., Wei X. (2024). Progress and Challenges of in Vivo Flow Cytometry and Its Applications in Circulating Cells of Eyes. Cytometry Pt A.

[12] Stepp M.A., Menko A.S. (2021). Immune Responses to Injury and Their Links to Eye Disease. Transl. Res..

[13] Park L.M., Lannigan J., Jaimes M.C. (2020). OMIP-069: Forty-Color Full Spectrum Flow Cytometry Panel for Deep Immunophenotyping of Major Cell Subsets in Human Peripheral Blood. Cytometry Pt A.

[14] Galindo-Cabello N., Sobas-Abad E.M., Lapresa R., Agulla J., Almeida Á., López A., Pastor J.C., Pastor-Idoate S., Usategui-Martín R. (2025). The TP53 Arg72Pro Polymorphism Predicts Visual and Neurodegenerative Outcomes in Retinal Detachment. Cell Death Dis..

